# Effect of Number of Days to Ambulation on Postoperative Daily Activities in Patients with Type A Aortic Dissection

**DOI:** 10.1298/ptr.E10308

**Published:** 2025-05-21

**Authors:** Yutaro OHNISHI, Tsubasa YOKOTE, Kengo SHIRADO, Shota OKUNO, Kenta KAWAMITSU, Kazuki YAMAUCHI, Takatoshi NISHIMURA, Masaya TANAKA, Takayuki UCHIDA, Shoji KAWAKAMI

**Affiliations:** 1 Department of Rehabilitation, Aso Iizuka Hospital, Japan; 2 Department of Cardiovascular Surgery, Aso Iizuka Hospital, Japan; 3 Department of Cardiology, Aso Iizuka Hospital, Japan

**Keywords:** Type A aortic dissection, Ambulation, Activities of daily living, Acute care

## Abstract

Objectives: This study aimed to investigate the effect of the number of days from surgery to ambulation on activities of daily living (ADL) at discharge in postoperative patients with type A aortic dissection (TAAD). Methods: It included patients with a diagnosis of TAAD who were independent in ADL before the onset of symptoms. ADL was assessed using the Katz Index (KI), with a KI score of 6 points at discharge defining independence and less than 6 points classified as dependence. Patients were divided into 2 groups based on independence in ADL at discharge. Logistic regression analysis was performed with independence in ADL at discharge as the object variable and the number of days from surgery to ambulation as the explanatory variable. A receiver operating characteristic curve was constructed to calculate the cutoff value. Results: A total of 100 patients were included in the analysis. There was a significant difference in the number of days to ambulation between the 2 groups. Multiple logistic regression analysis revealed that the probability of being independent in ADL at discharge was significantly lower with more days to ambulation (odds ratio: 0.93, 95% confidence interval: 0.86–0.99, P < 0.035). The cutoff value for the number of days from surgery to ambulation for independence in ADL at discharge was 8 days (area under the curve: 0.64). Conclusions: In postoperative patients with TAAD, the longer the postoperative days to start ambulation, the more difficult ADL recovery may be, but the cutoff values need further validation.

## Introduction

Type A aortic dissection (TAAD) is a surgical emergency caused by an intimal tear of the aorta forming a false lumen in the ascending aorta.^1)^ Patients with TAAD have shown improved survival rates due to advancements in medical technology.^2)^ Surgical treatment is more frequent than conservative therapy,^3)^ with a high proportion of cases requiring emergency surgery.^4)^ Surgical invasiveness is greater in emergency surgeries than elective surgeries and is associated with poor postoperative prognosis.^5,6)^ With the aging population in Japan, the number of older postoperative TAAD patients is increasing, leading to reports that older patients experience decreased activities of daily living (ADL) and face difficulties after returning home.^7)^ Therefore, ADL recovery after TAAD surgery is an important issue.

Recent studies have shown that early ambulation after elective cardiac surgery contributes to postoperative ADL recovery,^8)^ decreased incidence of postoperative complications,^9,10)^ shortened hospital stays,^8,9,11)^ increased rate of home discharge, and reduced 30-day readmission rates.^11)^ Early ambulation in patients after cardiac surgery is of vital importance. Nevertheless, postoperative TAAD patients are likely to be unstable for a longer period of time than outlined in the Japanese guidelines for elective cardiac surgery, which stipulate that sitting on the bed and standing should be started 1–2 days postoperatively, and ambulation 2–3 days postoperatively. Delayed ambulation, according to the guidelines, may lead to deterioration of overall condition. To our knowledge, there have been no reports verifying the effect on ADL of the number of days from surgery to ambulation initiation in TAAD postoperative patients in recent years, despite the recommendation of early mobilization in the field of cardiac surgery due to patient aging and advancements in medical technology.

Therefore, the aim of this study was to investigate the impact of delayed ambulation on ADL in postoperative TAAD patients and to calculate the cutoff value of the number of days to predict independence in ADL at discharge.

## Methods

### Subjects

The subjects were consecutive patients admitted to Iizuka Hospital (Iizuka, Fukuoka, Japan) diagnosed with TAAD that underwent emergency open-heart surgery and were prescribed rehabilitation between April 2014 and March 2021. Inclusion criteria were patients with independent ADL before the onset of the disease based on information from the patient’s family. Exclusion criteria included patients who required assistance prior to admission and those who were unable to start ambulation postoperatively. Patients who died during hospitalization were also excluded because of the difficulty in measuring outcomes.

### Ethical considerations and funding disclosure

This study was conducted with sufficient consideration for the protection of subjects in accordance with the Helsinki Declaration. Approval was obtained from the Ethics Committee of Iizuka Hospital (Approval No. R20013). Informed consent information was posted on the Aso Iizuka Hospital website based on the protocol approved by the ethics committee using an opt-out method. This study did not receive funding.

### Study design

This study was a retrospective observational study using data obtained from the medical records of patients.

### Definition of ambulation initiation

Due to the retrospective nature of the current study, it was necessary to define a baseline for ambulation distance, but there were no clear criteria. Therefore, for the present study, data were collected by defining the ambulation distance baseline as “the number of days taken to ambulate 15 m for the first time postoperatively”, with reference to previous studies.^12)^ Postoperative ambulation was initiated by physical therapists. The progression from bed rest to ambulation after surgery followed protocols established in previous studies.^13–16)^ Rehabilitation was started the day after surgery at this hospital, with a joint range of motion exercises, muscle strengthening exercises, and ADL exercises. The intensive care team disseminated information at conferences regarding the patient’s general condition and subsequent transition to sitting, standing, and the performance of ambulatory movement. The hospital proceeded with its mobilization program based on the decisions taken at this conference. The cessation criteria for ambulation were based on the physician’s judgment and the criteria of the Japanese Circulation Society guidelines.^17)^ Following the initiation of ambulation exercises, the patient was transitioned to an exercise therapy program. This program encompassed both aerobic and ADL exercises, with the objective of enhancing physical functionality.

### Criteria for independence in ADL

ADL was measured using the Katz Index (KI).^18)^ The reliability and validity of the KI as an assessment method for ADL have been verified^19)^ and it is extensively^20)^ used worldwide.^21)^ There were 6 evaluation items: bathing, grooming, toileting, transferring, feeding, and dressing. For each item, 1 point was given for independence and 0 points for dependence. In this study, independence was defined as a total score of 6 points, and dependence was defined as a total score below <6 points. ADL was assessed by nurses on admission and at discharge, and before the onset of illness based on information obtained from the patient’s family on admission. Discharge home should be considered when ADL is independent at the time medical treatment is generally completed. If ADL is not independent, the decision to transfer home should be made in consultation with multidisciplinary staff, the patient, and the patient’s family.

### Other measurement variables

Age, sex, body mass index (BMI), presence of dementia diagnosis, length of hospital stay (days), Charlson Comorbidity Index (CCI)^22)^ at admission, residual dissection, postoperative complications (cerebrovascular, respiratory, cardiovascular, renal, infectious), surgical technique, surgery duration, intraoperative blood loss, cardiopulmonary bypass operation duration, aortic cross-clamping duration, Sequential Organ Failure Assessment (SOFA) score^23)^ (postoperative day 1), postoperative mechanical ventilation duration, preoperative blood data (estimated glomerular filtration rate [eGFR], serum albumin [Alb], left ventricular ejection fraction [EF]), discharge to original place of residence, number of days from surgery to ambulation initiation, and information on ADL before and after admission were obtained from medical records.

### Statistical analysis

Patients were first divided into 2 groups: patients with independent ADL on discharge, and patients with non-independent ADL on discharge. Patient characteristics, intraoperative factors, and the number of days until ambulation initiation were compared between the 2 groups using Wilcoxon rank-sum test for continuous variables and Fisher’s exact test for categorical variables. Multiple logistic regression analysis was used to examine the association between the number of days until ambulation initiation and ADL recovery, calculating the odds ratio (OR) and 95% confidence interval (95% CI). The explanatory variables included were age,^24,25)^ sex,^24,25)^ CCI,^25–27)^ surgery duration,^6)^ and SOFA score (on postoperative day 1),^23)^ and postoperative cardiovascular complications,^9)^ based on previous studies showing their association with the number of days until ambulation initiation and ADL.

Additionally, a cutoff value for the number of days until ambulation initiation to predict independence in ADL at discharge was calculated using receiver operating characteristic (ROC) curve analysis, with independence in ADL as the object variable and the number of days until ambulation initiation as the explanatory variable. The cutoff values were calculated using the Youden index.

Finally, a comparison of patient characteristics was carried out between 2 further groups: patients who met the cutoff value for the number of days to ambulation, and those that did not. Statistical analysis was performed using R software (version 4.1.0, R Foundation for Statistical Computing, Vienna, Austria) with a significance level of 5%.

## Results

### Subjects

Data for a total of 119 patients were extracted. All subjects had a KI of 6 points. Among them, 19 patients (13 died during hospitalization and 6 failed to start ambulation) met the exclusion criteria and the remaining 100 patients were included in the final analysis. The median age was 68 years (range 59–76 years), and 50 people (50%) were male; 51 patients (51%) were independent in ADL at discharge. Of the eligible patients, 54% were discharged to their original place of residence.

### Differences in characteristics depending on independence in ADL

Table 1 shows baseline comparisons between the 2 groups classified based on independence in ADL. There were significant differences between the 2 groups in age, sex, dementia diagnosis, length of hospital stay, CCI, residual dissection, postoperative cerebrovascular complications, postoperative respiratory complications, SOFA score on postoperative day 1, postoperative mechanical ventilation duration, and preoperative eGFR. The number of days to start ambulation was significantly shorter in the independent ADL group (P < 0.05). In the group whose ADL had not recovered at discharge, the number of patients with independence in each of the KI subcategories was 20 (41%) for Feeding, 11 (22%) for Dressing, 1 (2%) for Bathing, 11 (22%) for Transferring, 16 (33%) for Toileting, and 20 (41%) for Continence.

**Table 1.** Comparison between 2 groups classified by Activities of daily living

Characteristic			Activities of daily living		P-value^2^
		Overall	Non-independent,	Independent	
		N = 100	N = 49	N = 51	
Age		68 (59, 76)	71 (65, 82)	64 (57, 73)	**0.003**
Sex	Male	50 (50%)	19 (39%)	31 (61%)	**0.028**
BMI, kg/m^2^		24.1 (21.7, 26.4)	23.3 (20.5, 27.3)	24.4 (22.6, 25.4)	0.200
Dementia diagnosis	Yes	6 (6.0%)	6 (12%)	0 (0%)	**0.012**
Length of hospital stay, days		36 (26, 51)	42 (29, 60)	31 (24, 44)	**0.011**
CCI, %	CCI > 2	16 (16%)	13 (27%)	3 (6%)	**<0.001**
Residual dissociation	Yes	66 (66%)	27 (55%)	39 (76%)	**0.024**
Postoperative cerebrovascular complications	Yes	16 (16%)	15 (31%)	1 (2.0%)	**<0.001**
Postoperative respiratory complications	Yes	58 (58%)	34 (69%)	24 (47%)	**0.024**
Postoperative cardiovascular complications	Yes	55 (55%)	30 (61%)	25 (49%)	0.200
Postoperative renal complications	Yes	28 (28%)	18 (37%)	10 (20%)	0.057
Postoperative infection complications	Yes	32 (32%)	18 (37%)	14 (27%)	0.300
Surgical technique	Ascending aortic replacement	44 (44%)	24 (49%)	20 (39%)	0.500
	Total arch replacement	40 (40%)	17 (35%)	23 (45%)	
	Multiple surgery	16 (16%)	8 (16%)	8 (16%)	
Operation time, min		401 (345, 488)	391 (332, 480)	403 (355, 496)	0.500
Intraoperative bleeding volume, ml		2858 (1926, 4457)	2552 (1883, 4500)	3050 (2000, 4408)	0.700
Operating hours of artificial heart-lung machine, min		209 (167, 248)	196 (168, 247)	214 (169, 249)	0.500
Duration of aortic blockade, min		134 (95, 174)	113 (94, 173)	144 (102, 175)	0.200
Postoperative day 1 SOFA score, points		11.0 (9.0, 13.0)	11.0 (10.0, 13.0)	10.0 (7.5, 12.0)	**0.017**
Duration of ventilator use, days		3.0 (1.0, 5.0)	4.0 (2.0, 6.0)	2.0 (1.0, 4.0)	**0.010**
Estimated glomerular filtration rate, mL/min/1.73 m^2^		41 (32, 57)	35 (27, 47)	51 (36, 63)	**<0.001**
Albumin, g/dL		3.2 (3.0, 3.5)	3.3 (3.0, 3.5)	3.2 (3.0, 3.5)	0.800
Left ventricular ejection fraction, %		61 (57, 64)	61 (60, 65)	60 (55, 63)	0.100
Number of days until ambulation started, days		7 (5, 11)	9 (5, 16)	6 (4, 8)	**0.020**
Discharge to original place of residence	Yes	54 (54%)	9 (18%)	45 (88%)	<0.001
KI, points		6.0 (1.0, 6.0)	1.0 (0.0, 3.0)	6.0 (6.0, 6.0)	**<0.001**
KI-Feeding, %	Independent	71 (71%)	20 (41%)	51 (100%)	**<0.001**
KI-Dressing, %	Independent	62 (62%)	11 (22%)	51 (100%)	**<0.001**
KI-Bathing, %	Independent	52 (52%)	1 (2.0%)	51 (100%)	**<0.001**
KI-Transferring, %	Independent	62 (62%)	11 (22%)	51 (100%)	**<0.001**
KI-Toileting, %	Independent	67 (67%)	16 (33%)	51 (100%)	**<0.001**
KI-Continence, %	Independent	71 (71%)	20 (41%)	51 (100%)	**<0.001**

Median (IQR); n (%).

Wilcoxon rank sum test; Fisher’s exact test.

BMI, body mass index; SOFA, sequential organ failure assessment; CCI, Charlson Comorbidity Index; KI, When discharged from hospital Katz Index.

### Relationship between ADL at discharge and number of days until ambulation initiation after surgery

**Fig. 1.**
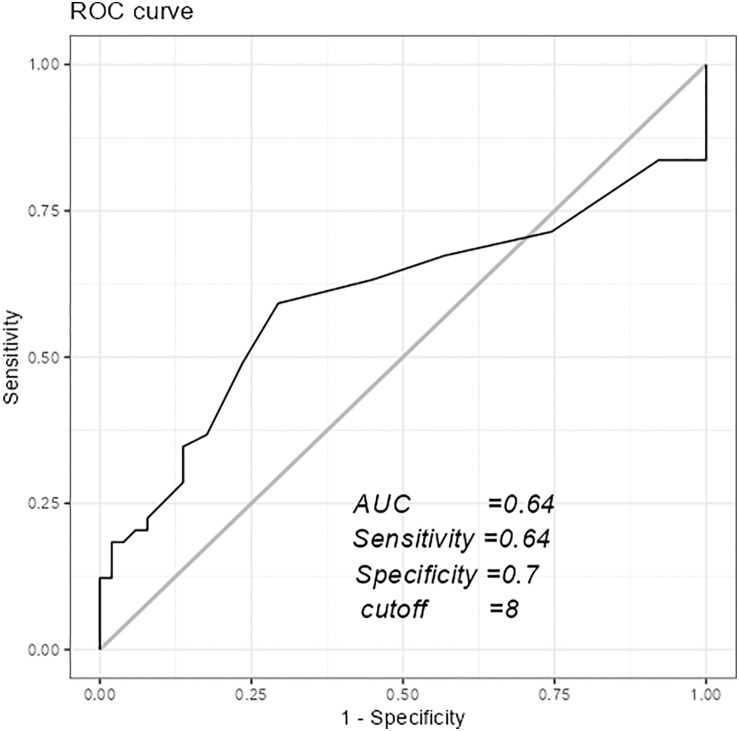
Receiver operator characteristic curve calculated using ADL and number of days until ambulation started. ROC, receiver operating characteristic; AUC, area under the curve, ADL, activities of daily living

Table 2 shows the results of multivariate logistic regression analysis. The odds ratio for ADL independence due to delay until ambulation initiation was 0.93 (95% CI = 0.86–0.99, P = 0.035). The cutoff value for the number of days until ambulation initiation to predict independence in ADL at discharge was 8 days, as shown in Figure 1. (area under the curve [AUC] 0.64, sensitivity 0.64, specificity 0.70).

**Table 2.** Relationship between ADL and the duration until ambulation through multiple logistic regression analysis

Characteristic	OR	95% CI	P-value
Number of days until ambulation started, days	0.93	0.86, 0.99	**0.035**
Age	0.96	0.91, 1.01	0.100
Female	0.38	0.13, 1.12	0.083
Operation time, min	1.00	1.00, 1.01	0.900
SOFA score (Postoperative day 1), points	0.89	0.72, 1.07	0.200
CCI > 2, points	0.22	0.04, 0.96	0.058
Postoperative cerebrovascular complications	0.05	0.002, 0.29	0.006

ADL, activities of daily living; SOFA, sequential organ failure assessment; CCI, Charlson Comorbidity Index; OR, odds ratio; CI, confidence interval

In the 2-group comparison between patients who met the cutoff value for the number of days to ambulation, and those that did not, length of hospital stay, postoperative infection complications, operation time, duration of aortic blockade, postoperative day 1 SOFA score, duration of ventilator use, left ventricular ejection fraction, discharge to original place of residence, KI, KI-Feeding, KI-Dressing, KI-Bathing, KI-Transferring, KI-Toileting, and KI-Continence were significantly different (Table 3).

**Table 3.** Comparison between 2 groups classified by days until start ambulation

Characteristic			Ambulation		
		Overall, N = 100	More than 8 days, N = 47	Less than 8 days, N = 53	P-value^2^
Age		68 (59, 76)	67 (59, 73)	69 (58, 76)	0.400
Sex	Male	50 (50%)	24 (51%)	26 (49%)	0.800
BMI, kg/m^2^		24.1 (21.7, 26.4)	23.9 (21.6, 27.1)	24.3 (22.2, 25.5)	>0.900
Dementia diagnosis		6 (6.0%)	5 (11%)	1 (1.9%)	0.100
Length of hospital stay, days		35 (26, 51)	44 (32, 58)	30 (23, 41)	**0.002**
CCI, %	CCI>2	16 (16%)	8 (17%)	8 (15%)	0.800
Residual dissociation	Yes	66 (66%)	31 (66%)	35 (66%)	>0.900
Postoperative Cerebrovascular complications	Yes	16 (16%)	11 (23%)	5 (9.4%)	0.057
Postoperative respiratory complications	Yes	58 (58%)	32 (68%)	26 (49%)	0.054
Postoperative cardiovascular complications	Yes	55 (55%)	28 (60%)	27 (51%)	0.400
Postoperative renal complications	Yes	28 (28%)	16 (34%)	12 (23%)	0.200
Postoperative Infection complications	Yes	32 (32%)	21 (45%)	11 (21%)	**0.010**
Surgical technique	Ascending aortic replacement	44 (44%)	15 (32%)	29 (55%)	0.057
	Total arch replacement	40 (40%)	24 (51%)	16 (30%)	
	Multiple surgery	16 (16%)	8 (17%)	8 (15%)	
Operation time, min		401 (345, 488)	418 (381, 510)	388 (327, 456)	**0.031**
Intraoperative bleeding volume, ml		2859 (1,927, 4,457)	3173 (2,054, 5,491)	2572 (1,846, 3,995)	0.066
Operating hours of artificial heart-lung machine, min		209 (167, 249)	220 (174, 256)	196 (160, 241)	0.091
Duration of aortic blockade, min		135 (95, 175)	149 (99, 180)	110 (92, 163)	**0.024**
Postoperative day 1 SOFA score, points		11.0 (9.0, 13.0)	12.0 (9.5, 13.0)	10.0 (8.0, 12.0)	**0.004**
Duration of ventilator use, days		3.0 (1.0, 5.0)	5.0 (3.0, 6.0)	2.0 (1.0, 3.0)	**<0.001**
Estimated glomerular filtration rate, mL/min/1.73m^2^		41 (32, 58)	38 (27, 52)	47 (32, 63)	0.085
Albumin, g/dL		3.2 (3.0, 3.5)	3.2 (3.0, 3.5)	3.2 (3.0, 3.6)	0.200
Left ventricular ejection fraction, %		61 (57, 64)	62 (60, 65)	60 (56, 62)	**0.032**
Number of days until ambulation started, days		7 (5, 11)	13 (10, 21)	5 (4, 6)	**<0.001**
Length of hospital stay after start ambulation, days		23.0 (17.0, 33.0)	22.0 (17.5, 36.5)	24.0 (17.0, 33.0)	0.860
Discharge to original place of residence	Yes	46 (46%)	31 (66%)	15 (28%)	**<0.001**
KI, points		6.0 (1.0, 6.0)	2.0 (0.0, 6.0)	6.0 (5.0, 6.0)	**<0.001**
KI-Feeding, %	Independent	71 (71%)	26 (55%)	45 (85%)	**0.001**
KI-Dressing, %	Independent	62 (62%)	20 (43%)	42 (79%)	**<0.001**
KI-Bathing, %	Independent	52 (52%)	15 (32%)	37 (70%)	**<0.001**
KI-Transferring, %	Independent	62 (62%)	19 (40%)	43 (81%)	**<0.001**
KI-Toileting, %	Independent	67 (67%)	23 (49%)	44 (83%)	**<0.001**
KI-Continence, %	Independent	71 (71%)	26 (55%)	45 (85%)	**0.001**

Median (IQR); n (%).

Wilcoxon rank sum test; Pearson’s Chi-squared test; Fisher’s exact test.

BMI, body mass index; SOFA, sequential organ failure assessment; CCI, Charlson Comorbidity Index; KI, When discharged from hospital Katz Index

## Discussion

This study is the first to elucidate the impact of the time from surgery to ambulation on ADL recovery in patients with TAAD. However, predicting ADL independence based on an 8-day cutoff has limitations, as indicated by the AUC of 0.64, suggesting that the predictive accuracy is not particularly high. Furthermore, recovery of ADL may be influenced by the degree of improvement in ADL other than ambulation, which requires careful interpretation.

The difficulty in ADL recovery with a longer time to ambulation initiation was consistent with previous studies on other elective cardiac surgery patients.^8)^ In our study, the group with non-recovered ADL had a significantly longer mechanical ventilation duration. Prolonged mechanical ventilation in cardiac surgery patients has been associated with increased complication rates due to prolonged bed rest, potentially leading to neurological and muscular deterioration.^28,29)^ Furthermore, the delay in initiating ambulation, which is effective in preventing muscle weakness by activating major muscles and stimulating respiratory improvement and peripheral muscle contraction,^30)^ may hinder ADL recovery. In our hospital, rehabilitation is started even before TAAD patients start ambulation. After they began to ambulate, aerobic exercise and resistance training were performed to increase their activity level. The results of this study showed no significant difference in the number of days in hospital after starting ambulation, making it unlikely that non-independence on ADL was due to the length of hospital stay with appropriate rehabilitation. Early start ambulation may be an important factor in the recovery of ADL, as ADL declined even in TAAD patients who received adequate exercise therapy. As demonstrated by Igarashi et al.,^7)^ postoperative complications have been shown to affect mortality and home discharge rates. In this study, the non-independent group exhibited a significantly higher complication rate, suggesting that these complications may have affected ADL at discharge. Postoperative cerebrovascular complications,^31)^ a poor prognostic factor after cardiac surgery, were shown to be a significant independent factor affecting ADL decline in this study. However, even after accounting for this influence, the number of days of start ambulation was still an independent factor for ADL. Consequently, even if ambulation is feasible at an early stage, if postoperative complications emerge, the prognosis should be evaluated promptly.

The cutoff value predicting independent ADL at discharge was 8 days, longer than the recommended ambulation initiation of 2–3 days for elective patients undergoing valve replacement or coronary artery bypass surgery.^17)^ Given the substantial surgical trauma and severity of postoperative conditions in TAAD patients,^32)^ factors delaying ventilator weaning might have extended the period until overall stability was achieved, potentially prolonging the duration until ambulation. The number of days to start ambulation was also longer than the median time (day 5) in previous studies of TAAD.^33)^ The previous study was conducted in an exploratory study, and the patient background differs from this one, making an exact comparison difficult. However, it was consistent with our findings that starting ambulation as early as possible impacted ADL recovery.”

The study showed that an AUC of 0.64, although not particularly predictive, may indicate a potential return to independent ADL if ambulation is initiated within 8 days postoperatively. The validity of the cutoff values calculated in this study needs further verification. Patients who needed more than 8 days to start ambulation were characterized by a longer operating time and tended to have a higher severity of illness the day after surgery. In cases with these factors, prevention of disuse syndrome with multidisciplinary intervention may be necessary from the early postoperative period. In addition, it may be difficult for patients to return to their original place of residence if they start ambulation after more than 8 days, so it is important to share information with multidisciplinary staff and the patient’s family and discuss future policies from an early stage.

### Limitations

There are several limitations to this study. First, the small sample size necessitates caution in generalizing the results, warranting larger studies in the future. Second, being a single-center study, there may be biases in patient backgrounds and factors, necessitating validation in large, multi-center populations. Third, the data used in this study were retrospectively extracted, potentially leading to inadequate collection of necessary information, warranting prospective longitudinal studies in the future. In this study, ADL was assessed prior to admission, but a detailed physical function assessment could not be carried out. Even if patients are able to start ambulation early after surgery, those with poor pre-admission physical function may be more susceptible to surgical invasion and postoperative complications, which may make it more difficult for them to recover ADL at discharge. The possibility that TAAD patients with characteristics that make it difficult to recover ADL may have taken longer to start ambulation cannot be ruled out and needs to be verified in the future.

## Conclusions

In TAAD patients, the time from surgery to ambulation initiation influences ADL at discharge, with the cutoff value for predicting independent ADL being 8 days.

## Author’s contributions

Tsubasa Yokote contributed to the writing of the paper and the research design. Kengo Shirado and Shota Okuno contributed to the writing of the paper. Kenta Kawamitsu contributed to the study design and to the determination and execution of the statistical analysis. Kazuki Yamauchi contributed to data collection. Takatoshi Nishimura and Masaya Tanaka contributed to proofreading the paper. Shoji Kawakami contributed to initiating and revising the study. Takayuki Uchida contributed to the planning of the study and critical revision of the paper.

## Funding

Not applicable.

## Conflict of Interest

There are no conflicts of interest to disclose.
